# Diverticula in Male *Lycorea halia* Butterflies (Lepidoptera: Nymphalidae: Danaini: Itunina)—Support Organs for Everted Hairpencils with Unique Ultrastructure

**DOI:** 10.1007/s13744-019-00720-6

**Published:** 2019-12-05

**Authors:** W Gnatzy, O W Fischer, A Kiesel, R I Vane-Wright, M Boppré

**Affiliations:** 1 Institut für Ökologie, Evolution and Diversität, Goethe Universität, Frankfurt am Main, Germany; 2 Forstzoologie und Entomologie, Albert-Ludwigs-Universität, 79085 Freiburg im Breisgau, Germany; 3 Dept of Entomology, The Natural History Museum, London, Durrell Institute of Conservation and Ecology (DICE), and School of Anthropology and Conservation, Univ of Kent, Canterbury, UK

**Keywords:** Androconial organs, functional morphology, extracellular fibril bundles, courtship

## Abstract

The involvement of the diverticula, a synapomorphy for Itunina, in protrusion and expansion of hairpencils by male *Lycorea halia* (Hübner, 1816) is demonstrated for the first time. They facilitate maintaining the haemolymph pressure necessary to keep the hairpencils everted. The diverticula are curved hook-like lobes, open to the body cavity and densely filled with tracheae and threads made by units of two staggered cells surrounding a central extracellular fibril bundle. Such complex structures, apparently metabolically active, have not been reported for insects previously and might indicate additional functions, but their functional role(s) remains a puzzle. When a male emerges from pupa, the diverticula are not yet formed; this happens only during the first protrusion of the hairpencils.

## Introduction

Milkweed butterflies (Nymphalidae: Danainae: Danaini) of the genera *Lycorea* and *Anetia* (Itunina) share unique structures associated with the male genitalia and the androconial organs (hairpencils). Named the ‘dorsal diverticulum’ by Ackery and Vane-Wright (1984:27), these organs represent a pair of hook-shaped lobes, first depicted by D’Almeida (1939: plate 12, 14) but without any comment. In general, males of milkweed butterflies exhibit a wide variety of specialisations associated with chemical communication (Ackery & Vane-Wright 1984, Boppré & Vane-Wright 1989) but nothing comparable to the diverticula. So far, diverticula have only been seen in dry-preserved butterflies from museum collections (D’Almeida 1939: plate 12, fig. 14, Ackery & Vane-Wright 1984). Here, we report on the use of these organs by living males and their morphology and ultra-structure.

## Material and Methods

Pupae of *Lycorea halia* (Hübner, 1816) were purchased (as *L. cleobaea*) from dealers; they originated from captive breeding in Belize, El Salvador and Costa Rica. Butterflies were kept in gauze cages and fed with a sugar-honey solution.

### Live observations

To study the use of diverticula and hairpencils, males were mechanically irritated by holding them by their wings. Overview images and video clips of protruding and everted hairpencils were taken with a Nikon Coolpix 4500.

### Light microscopy

For observing fresh diverticula and hairpencils and for dissecting abdomens, males of different age were killed in a deep freezer and a stereo microscope (ZEISS SteREO Discovery.V8) was employed. Images were obtained with a KEYENCE VHX-700FD digital microscope equipped with a VH-Z20R/VH-Z20W zoom lens 20–200× and a polarisation filter OP-87429. Several abdomens were fixed with 25% glutardialdehyde: 0.1 M Na cacodylate (1:3) and stored in 70% ethanol for later study, some were macerated in KOH, and methylene blue was applied for getting better contrast.

### Scanning electron microscopy

For scanning electron microscopy (SEM), hairpencils and diverticula were either air dried or (mostly) treated after Nation (1983), glued on aluminium stubs using adhesive carbon disks (Leit-Tab; Plano GmbH, Wetzlar, Germany), gold-coated in an Edwards Sputter Coater and studied at an accelerating voltage of 10 kV with a ZEISS DMS 940A equipped with a DISS5 unit (point electronic; Halle, Germany).

Preparations for field emission scanning electron microscopy (FeSEM) were prefixed as for transmission electron microscopy (TEM) (below). After that, the specimens were dehydrated and critical-point dried in a Polaron unit using CO_2_ and amyl-acetate (2 × 15min). The dried preparations were mounted with carbon disks (as above) or with minute drops of conductive carbon cement (Leit-C; Plano, Wetzlar, Germany) on aluminium stubs (as above). Finally, the specimens were coated with gold in an Edwards Sputter Coater and then examined using a Hitachi S-4500 FeSEM at an accelerating voltage of 1–5 kV.

### Transmission electron microscopy

Freshly isolated diverticula were fixed at 4°C in a primary fixative of 2% glutardialdehyde dissolved in a rinse buffer of 0.16 M cacodylate (pH 7.2–7.3) containing 10% sucrose. After 2*.*5 h, tissues were washed three times with rinse buffer before being transferred to a second fixative (2% osmium tetroxide dissolved in rinse buffer, w/v) for 2 h. After thoroughly washing with rinse buffer, tissues were gradually dehydrated in a graded ethanol series (30–100% v/v). From absolute ethanol, specimens were transferred to propylene oxide and infiltrated with mixtures of propylene oxide and Epon 812 before embedding in pure Epon resin. Ultrathin silver to golden sections (60–80 nm) were made using diamond knives (DuPont) on an Ultracut E ultramicrotome (Reichert-Jung, Wien, Austria), mounted on Pioloform-coated slot grids and double-stained with uranyl acetate and Luft’s lead citrate to enhance contrast. Images were taken with a Phillips CM 12 transmission electron microscope operating at 80 kV.

### Documentation

Digital images were adjusted for brightness and contrast with Adobe® Photoshop® CS6 and compiled with Adobe® InDesign® CS6 on a MacBook Pro.

## Results

### Hairpencils and diverticula

**Fig 1:**
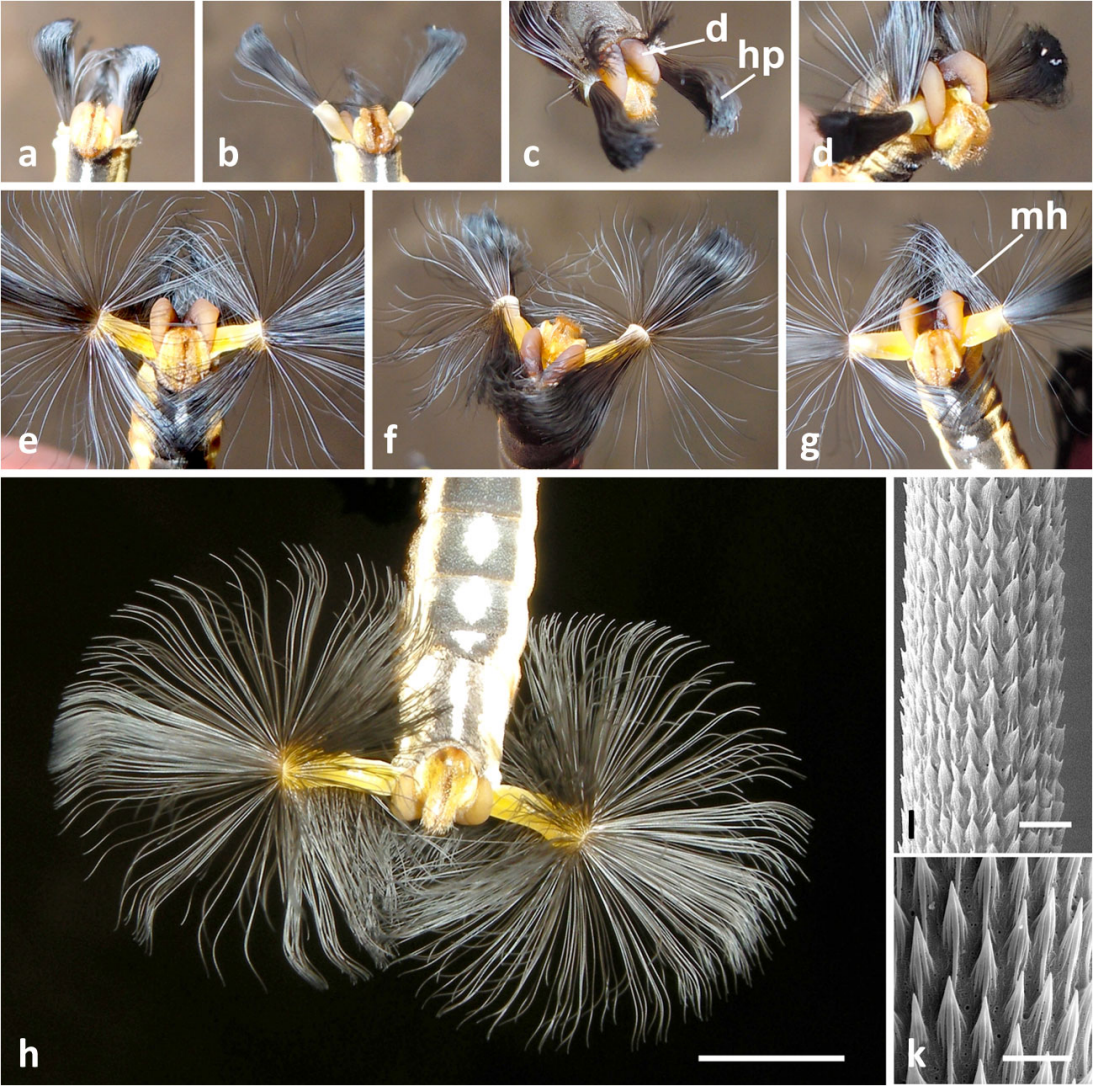
Macrophotographs of hairpencils and diverticula in *Lycorea halia*: (A–G) protrusion of hairpencils (hp) and diverticula (d) by living males in different stages and views. (H) Fully expanded hairpencils, note diverticula latching onto the hairpencil stalks. (I, K) SEM of a hairpencil hair. (A, B, E, G, H) Ventral, (C, D) lateral, (F) dorsal, view. *mh* ‘mohican’-like hair crest. Scale bars: H 5 mm, I 10 μm, K 5 μm.

When one catches a male *Lycorea* butterfly, it will instantly expand its hairpencils (see Discussion). With haemolymph pressure (as in other Danaini; Pliske & Salpeter 1971), the hairpencil sheaths are everted like turning a finger of a glove inside out, and the hairs unfold successively. This also happens when holding a male by its wings only. Eventually, the hairs form large spheres of impressive appearance (Fig 1H). Along the hairpencil tubes, a dorsal crest of long hairs is apparent—reminiscent of a mohican haircut (Fig 1G).

Two curved lobes, the diverticula, appear simultaneously with the hairpencils at the tip of the abdomen (Fig 1A–G). When hairpencil hairs are fully expanded (Fig 1H), the diverticula show up dorsally as yellowish or yellowish-brown hooks with their tips directed downwards and enclosing the bases of the hairpencil sheaths (Fig 1B–H). As a result, the exerted diverticula bring the hairpencil sheaths laterally perpendicular to the long axis of the abdomen and support them in this position.

Hairpencil display can last for several minutes. When the insect is released, hairpencils and diverticula are withdrawn into the abdomen. Expansion can be elicited after a short pause again; however, eventually, the response decreases (habituation).

### Anatomy of hairpencils and diverticula

**Fig 2:**
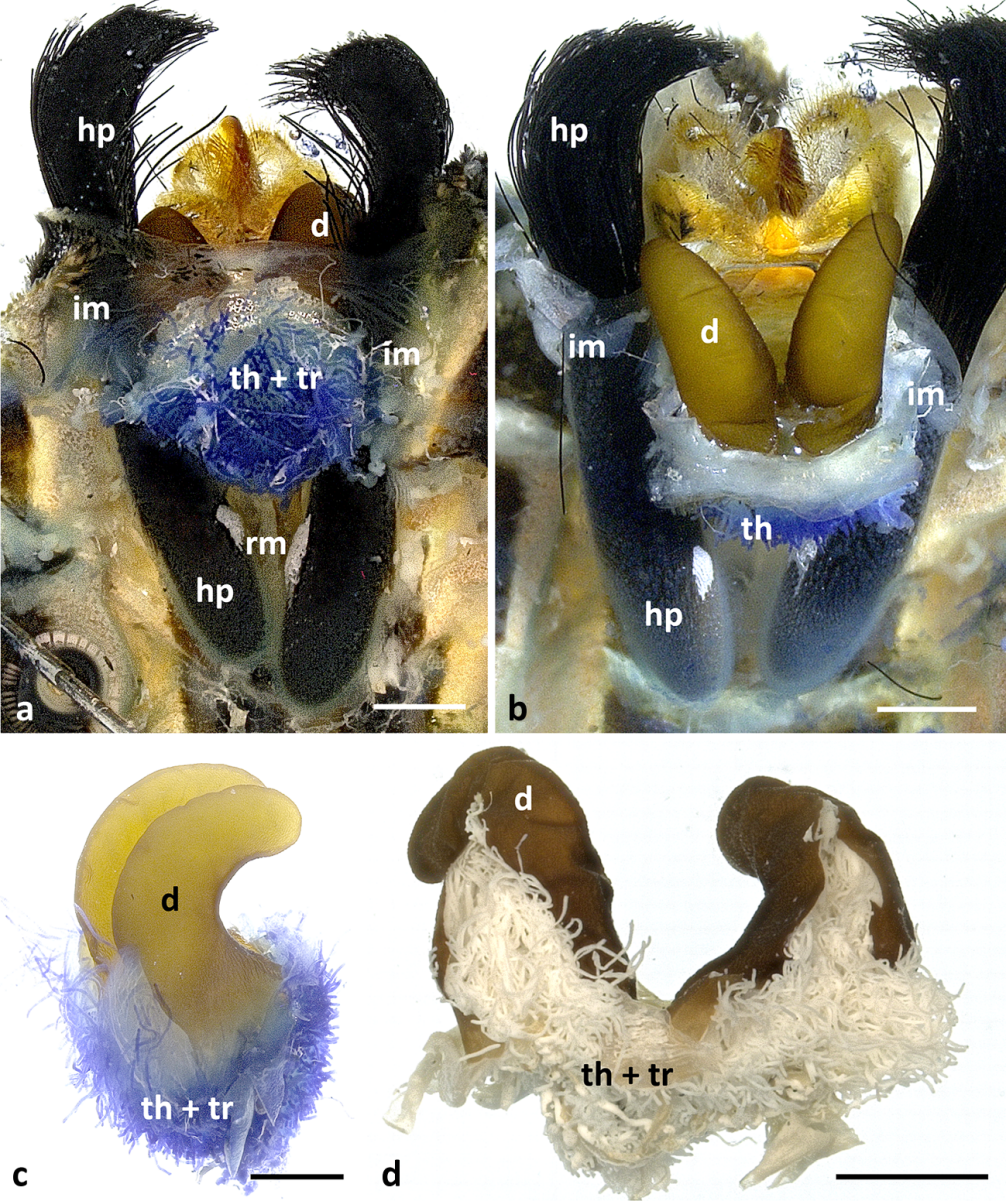
Anatomy of the abdominal tip and diverticula of male *Lycorea halia* in dorsal view: (A, B) overviews in different viewing angles showing diverticula (d), diverticula threads (th) and tracheae (tr), hairpencils (hp) and the intersegmental membrane VIII–IX (im); *rm* retractor muscle; compare with Fig 8A, B. (C) Isolated diverticula with threads sticking out, entangled by numerous tracheae. (D) Isolated diverticula slit open showing that threads and tracheae ooze out. Scale bars: 1 mm.

The hairpencil hairs insert at and rest within two sheaths with retractor muscles at their bases (Fig 2A, B). The number of hairs per brush is about 1,500, their length is ca 7 mm and their diameter ca 25 μm. The surface is evenly sculptured (Fig 1I, K).

In a dissected abdomen, the two 3.5-mm-long diverticula, which lie dorsal to the external genitalia between the hairpencil sheaths, are very conspicuous (Fig 2A, B). The intersegmental membrane between segments VIII–IX links the diverticula with the uncus as well as the hairpencil sheaths. Thus, protrusion and retraction of both hairpencils and diverticula is made possible (see below). In relation to the haemocoel, the diverticula are open to the lumen of the abdomen with a bunch of ca 18-μm-thick threads sticking out for up to 300 μm (Figs 2 and 4).

### Ultrastructure of diverticula

**Fig 3:**
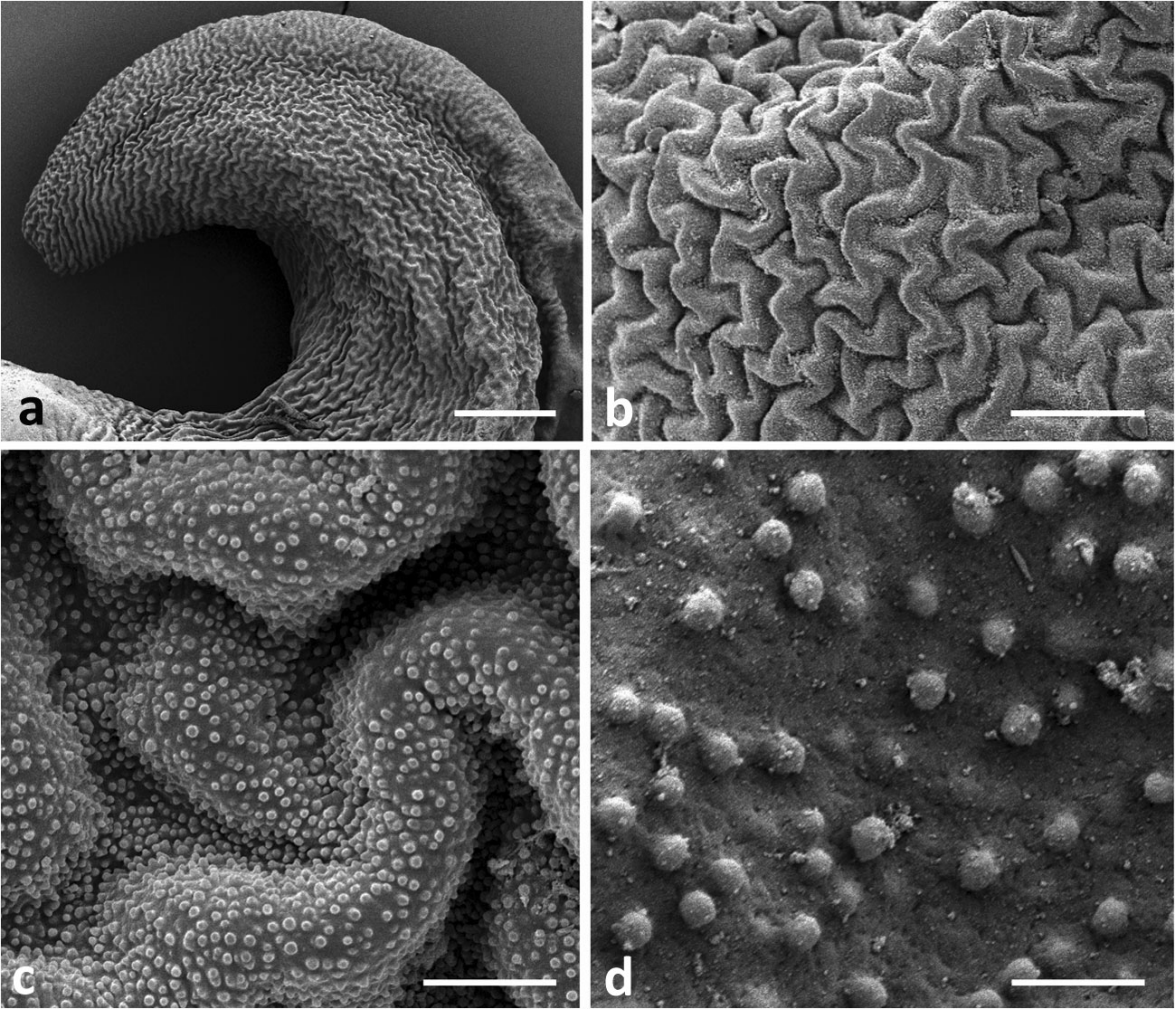
FeSEM (A, D) and SEM (B, C) images of the surface of diverticula in *Lycorea halia* in increasing magnification. Scale bars: A 200 μm, B 50 μm, C 10 μm, D 2 μm.

**Fig 4:**
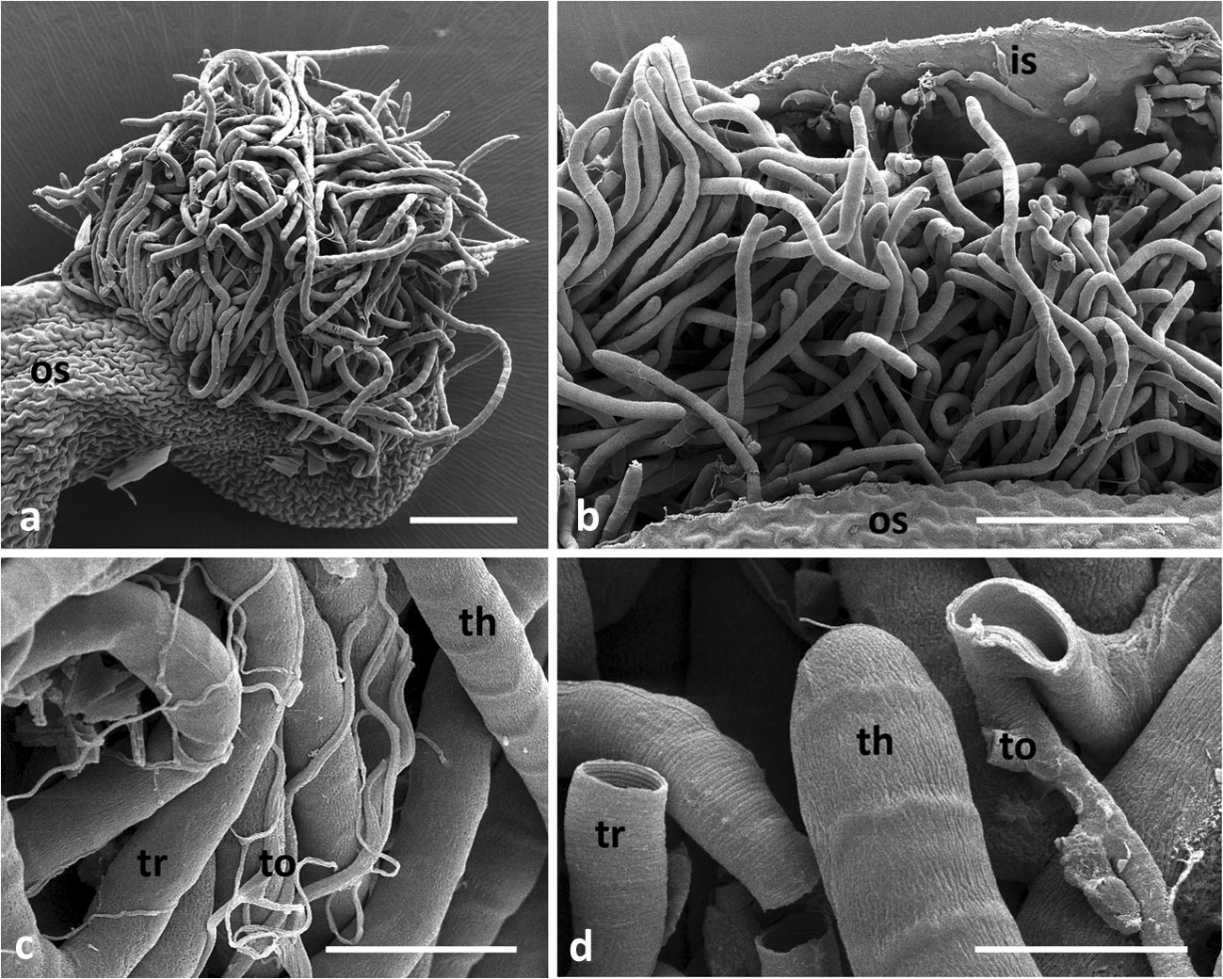
SEM images of diverticula threads (th) and tracheae (tr) in slit open diverticula of *Lycorea halia*: (A, B) threads oozing out a diverticulum. (C, D) Threads and tracheae and tracheoles. *is* inner surface of diverticulum where threads originate, *os* outer surface of diverticulum (see Fig 3), *to* tracheole. Scale bars: A, B 200 μm, C 40 μm, D 20 μm.

The surface of diverticula shows a rugose appearance (Fig 3), at higher magnification, a folded, granular structure, with roughly spherical extrusions about 7 μm in diameter (Fig 3D). Inside, diverticula are densely packed with the threads (Figs 2D and 4A, B), which originate at the inner wall of a diverticulum and end blindly (Fig 4B, D). They are surrounded by haemolymph and numerous tracheae and tracheoles (Figs 2 and 4C, D).

**Fig 5:**
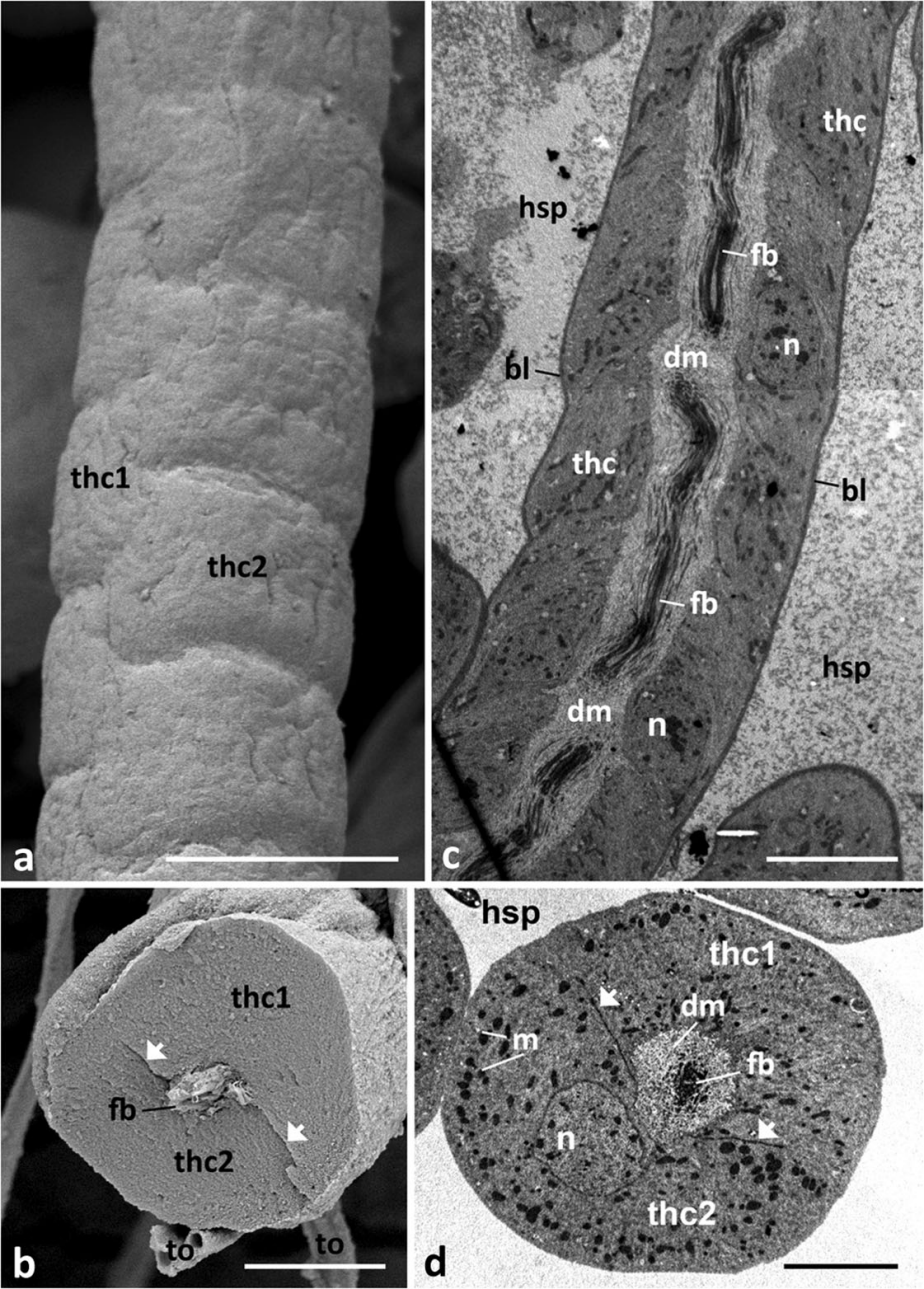
Ultrastructure of threads of diverticula in *Lycorea halia*: (A, B) under FeSEM, (C, D) under TEM. (A) Outer surface showing thread units, (B) cross fracture, (C) longitudinal and (D) cross section. *Arrows* cell boundaries, *bl* basal lamina, *fb* fibril bundle, *dm* diffuse material, *m* mitochondria, *n* nucleus, *hsp* haemolymph space, *thc1*, *thc2* the two cells which form a thread unit, *to* tracheole. Scale bars: A, C 10 μm, B, D 5 μm.

**Fig 6:**
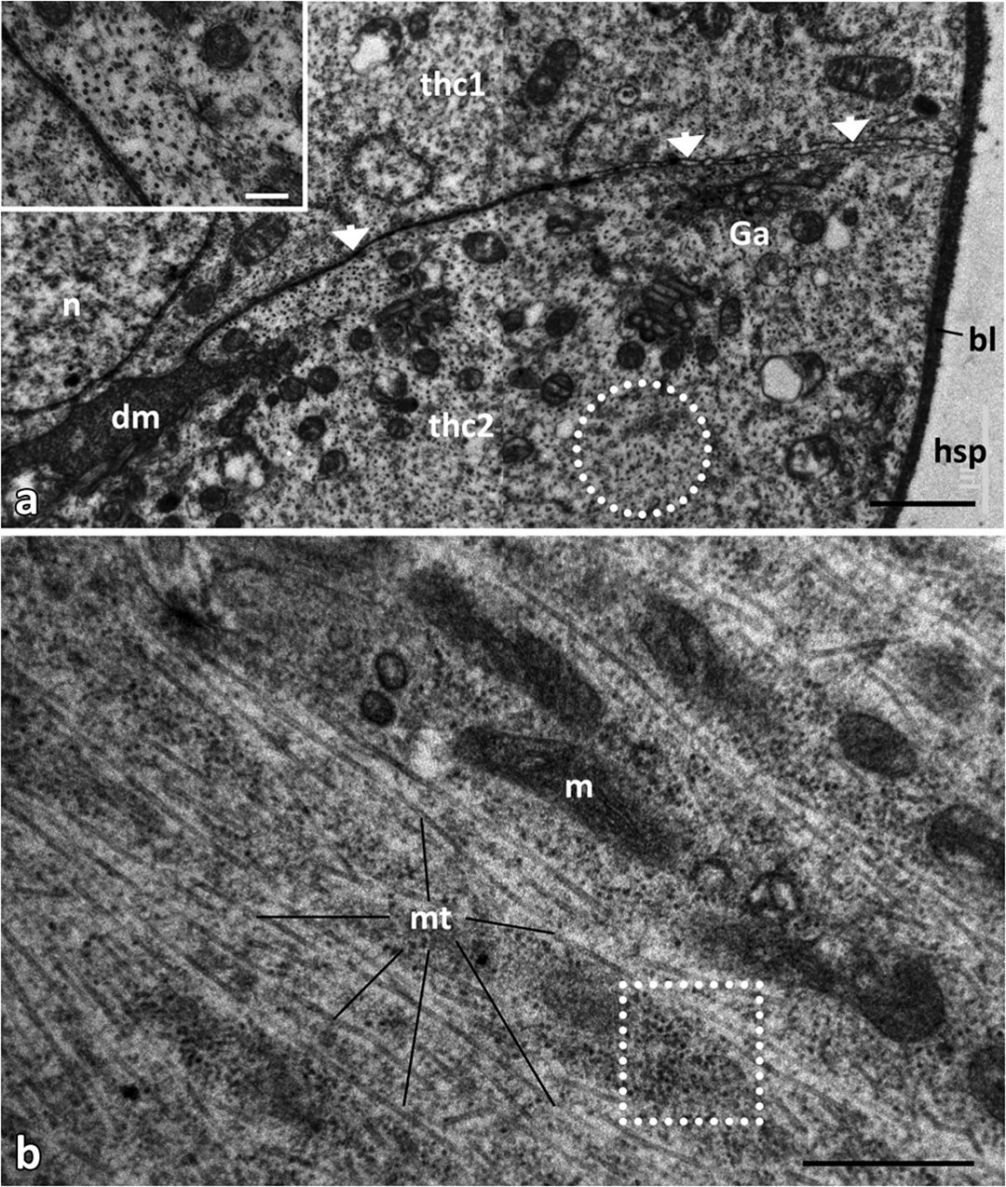
Cytoplasmic organisation of thread cells in male *Lycorea halia*: (A) TEM cross-section through a single thread showing cell boundaries between thread cells (thc1, thc2); inset microtubules (arrows) enlarged. (B) TEM longitudinal section through a thread cell. *Arrow heads* cell boundary with desmosomes, *bl* basal lamina, *dotted circle* cross-cut microtubules, *dotted square* free ribosomes, *dm* diffuse material, *Ga* Golgi apparatus, *hsp* haemolymph space, *m* mitochondria, *mt* microtubules, *n* nucleus. Scale bars: A 1 μm, inset A 0.2 μm, B 0.5 μm.

**Fig 7:**
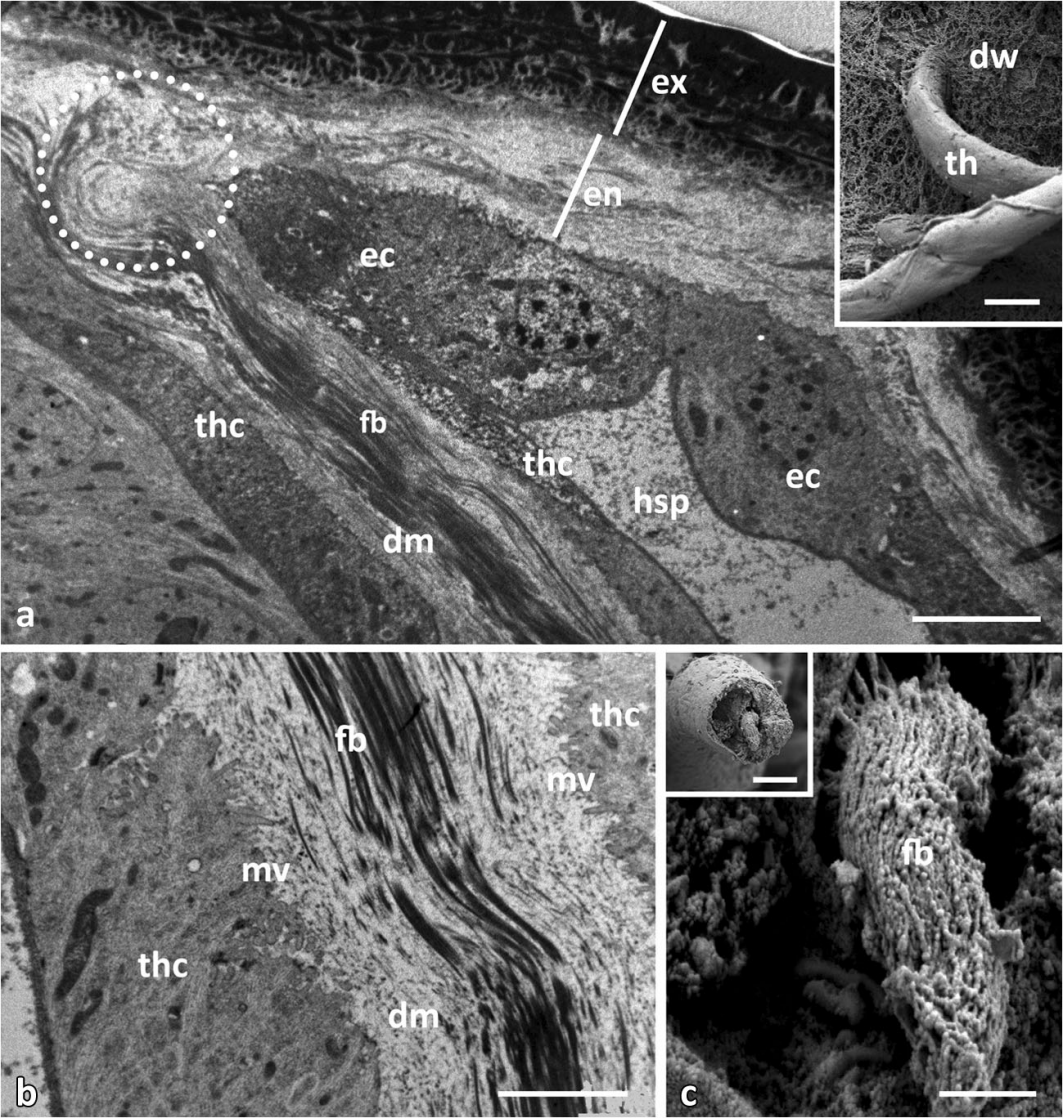
Details of diverticula threads of *Lycorea halia*: (A) anchoring of a thread (th) via its fibril bundle (fb) in the endocuticle (en) of a diverticulum wall. (B) Fibril bundle (fb) within diffuse material (dm) and microvilli (mv) at cell border. (C) Fibril bundle of a cross fractured thread. (A, B) longitudinal TEM sections, insets (A and C) FeSEM. *Dotted circle* anchoring area, *dw* diverticulum wall, *ec* epidermal cell, *ex* exocuticle, *hsp* haemolymph space, *mv* microvilli, *thc* thread cell. Scale bars: A, inset A 10 μm, B, C 1 μm, inset B 5 μm.

The ultrastructure of the threads is unique. Each consists of countless units stacked in a cylindrical column, like a rouleau (Fig 5A). A thread unit is approx. 10 × 7 μm and is made up by two cells which are arranged more or less crescent-shaped, lining an extracellular space in their centre (Fig 5B, D). Each thread cell is separated from the haemolymph space by a prominent basal lamina (Figs 5C and 6A); thread units are arranged slightly staggered (Fig 5A). The apical cell membrane to the extracellular space is differentiated into short microvilli (Fig 7B). The two cells forming a unit are inter-connected via zonulae adhaerentes; these cell connections exhibit septate desmosomes (Fig 6A cmp. with 5B, D).

All thread cells possess spherical, well-developed nuclei measuring about 5–6 μm with a prominent nucleolus and decondensed chromatin. Their cytoplasm houses numerous microtubules and mitochondria (Figs 5D and 6B) which are characterised by transversally oriented cristae and a dense matrix. The number of free ribosomes is remarkable (Fig 6B). A weakly developed tubular form of endoplasmic reticulum can be found throughout the cytoplasm.

All cells making a thread form an extracellular space running centrally along its longitudinal axis (Figs 5C and 7A, B) which contains fibril bundles surrounded by a matrix of diffuse material. The fibrils show up in TEM sections (Figs 5C, D and 7A, B) but also in SEM (Figs 5B and 7C). Probably, the fibrils represent a continuous string throughout the entire length of the canal but this could not be verified with certainty. Each thread is anchored at the diverticulum endocuticle by its fibril bundle (Fig 7A).

### Formation of diverticula during adult life

During adult life, the diverticula change their colour from fleshy white-yellow to dark brown, likely due to sclerotisation.

**Fig 8:**
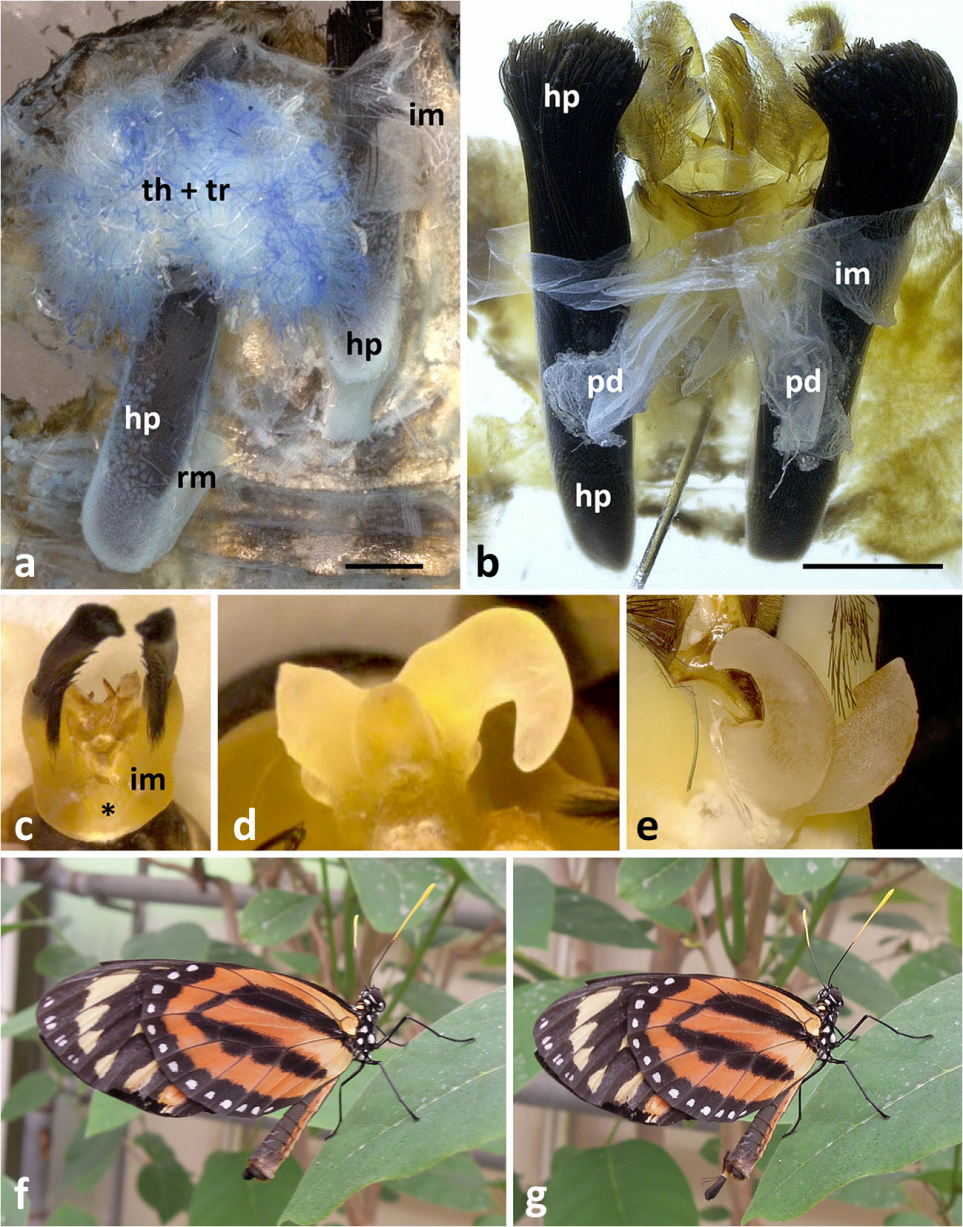
Anatomy of the abdominal tip of a *Lycorea halia* male in the state of emerging from pupa: (A) two bundles of threads (th, blue), in between many shiny tracheae (tr, white), hairpencil sheaths (hp), retractor muscles (rm) and intersegmental membrane VIII–IX (im). (B) Preparation as in A but treated with KOH, showing intersegmental membrane of proto-diverticula (pd) without (except small residuals) threads; note the proto-diverticula are inverted; compare with Fig 2A, B. (C) Hairpencil tips plus intersegmental membrane appearing due to artificial protrusion, with diverticula not yet protruded; viewed ventrally; the asterisk marks from where they will appear, compare with Fig 1. (D, E) Intermediate state of diverticula formation. (C–E) Grabbed from video clips. (F, G) Male protruding hairpencils partially. Scale bars: 1 mm.

Dissections of males in the process of emerging from pupa which thus had never before protruded their hairpencils show a very different condition than that of older males (see above): no diverticula are visible, instead there is a large bunch of threads (Fig 8A), attached to the yet flexible intersegmental membrane VIII–IX. Treatment with KOH dissolves the threads but leaves the membrane (Fig 8B). Thus, from these proto-diverticula, the diverticula are formed when the hairpencils are protruded for the first time. Indeed, when the abdomen of a newly emerged male is squeezed from front to back under a microscope, it can be seen that the diverticula appear like eversion of a finger of a glove (Fig 8D, E), similar to the hairpencils—the threads are inside. Diverticula then show up as light lobes, yet missing their final hook-like shape (Fig 8E). Thus, in an emerging male, the proto-diverticula lie within the haemocoel and via an adult behaviour (first protrusion), they are packed into sheaths (part of the intersegmental membrane); these sclerotise and henceforth retain their form as stable hooks which are protruded and retracted jointly with the hairpencils.

Figures 8F and 8G show a male protruding its hairpencils in part, never fully, while sitting on a leaf. They are from a series of photographs taken in a butterfly house in ignorance of the unusual formation of the diverticula—perhaps, they document a male protruding the hairpencils for the first time for forming its diverticula.

## Discussion

### Hairpencil employment in Danaini

Courtship behaviour of milkweed butterflies involves the display of male hairpencils for dissemination of pheromones in close vicinity to a female (Brower *et al*^1965^, Boppré 1984, Boppré & Vane-Wright 1989). *Lycorea ceres* (D’Almeida 1939) is the species for which male scents have been documented and analysed for the first time in Lepidoptera (Meinwald *et al*^1966^). As yet no field-studies on the behaviour of this taxon have been reported although collectors know that *Lycorea* expand their hairpencils when being handled (DeVries 1987, Meinwald *et al*^1966^, Hernández-Baz *et al*^2019^), and DeVries (1987:213) stated: ‘Males perch during the morning in the sub-canopy ... occasionally with the hair pencils extruded’.

Courtship in Danaini has rarely been analysed in detail. The study by Brower *et al* (1965) on the queen butterfly, *Danaus gilippus* (Cramer 1776), remains the most complete, while the one by Pliske (1975) deals with *Danaus plexippus* (Linnaeus 1758) which is not representative with respect to courtship in Danaini (Boppré 1993, Brower *et al*^2007^). The hairpencils of many Danaini disseminate pheromone-transfer-particles (PTPs; Boppré & Vane-Wright 1989) during courtship and are expanded only briefly (maximally for some seconds) close to a female’s antennae. On the contrary, *Euploea* species, none of which employs PTPs, have been seen with expanded hairpencils not only when hovering above a female but also when flying in ‘courtship mood’ with no female in their vicinity (Latter & Eltringham 1935, Sevastopulo 1944, Hardy 1951), then disseminating pheromones, perhaps, to attract a female, to repel other males or to mark a territory? On the courtship of *Lycorea*, relatively rarely encountered forest butterflies, we can only guess. For several reasons, we assume it is similar to *Euploea*: DeVries (1987) saw males flying with expanded hairpencils; both taxa expand their organs voluntarily when mechanically stimulated (perhaps to startle predators upon attack); *Lycorea* and *Euploea* are also quite close with respect to their larval hostplants being the only danaine genera using species of Moraceae (*Ficus* spp.) (Ackery & Vane-Wright 1984, cf. Boppré *et al*^2020^). Unfortunately, we had the experience that *Lycorea* do not court and mate in flight cages as, e.g. *Euploea* do (Boppré *et al* unpubl.). Our finding that the diverticula are voluntarily protruded together with the hairpencils (which has not been observed before) and latch at the bases of the hairpencil stalks suggests that the diverticula are support organs which facilitate maintaining the haemolymph pressure necessary to keep the hairpencils everted. Perhaps, *Lycorea* fly for longer periods and/or more often with their hairpencils expanded than *Euploea*?

### Ultrastructural and developmental peculiarities of diverticula

As supporting organs for expanded hairpencils during courtship activities, simple cuticular hooks would do. However, the architecture of the diverticula is most complex, exhibiting unexpected ultrastructural details and the organs appear to be metabolically active. Perhaps, their primary function is all but a hook―but if so, what other function could this extraordinary organ have? It may be significant that the related genus *Anetia* has what appears to be comparable paired structures that are much smaller and would not seem capable of providing a similar mechanical function.

Other Danaini in addition to hairpencils possess alar androconial organs with which mechanical contacts of the hairpencils are established behaviourally independent of courtship activities (Boppré & Vane-Wright 1989). *Lycorea* are devoid of alar organs―do diverticula contribute to the pheromones released by the hairpencils or to their biosynthesis? Then, however, intimate mechanical contact between diverticula and hairpencil hairs would be essential.

The surprising finding that diverticula achieve their final shape during their first protrusion by the adult male, adds to the puzzle. Or can this shed light onto the mystery and present the key for understanding diverticula fully? Perhaps, the unique threads primarily fulfil an important function during the pupal stage? However, to the best of our knowledge, such extracellular cell-shielded fibril bundles have never been described before—but we lack any idea regarding their functional role.

In any case, circumstancial evidence suggests that the diverticular threads are metabolically active: they are in connection with the haemolymph and the tracheal system, thread cells possess large nuclei, many mitochondria and numerous ribosomes. If only a physiological rôle could be recognised.

The biology of milkweed butterflies is less understood than one might assume in view of the huge amount of literature on this taxon (see Boppré *et al*^2019^), and our paper adds another dimension, namely usually invisible internal structural peculiarities.
